# Screening for Bipolar Disorder Symptoms in Depressed Primary Care Attenders: Comparison between Mood Disorder Questionnaire and Hypomania Checklist (HCL-32)

**DOI:** 10.1155/2013/548349

**Published:** 2013-04-07

**Authors:** Anna Sasdelli, Loredana Lia, C. Claudia Luciano, Claudia Nespeca, Domenico Berardi, Marco Menchetti

**Affiliations:** Institute of Psychiatry, University of Bologna, Viale C. Pepoli 5, 40123 Bologna, Italy

## Abstract

*Objective*. To describe the prevalence of patients who screen positive for bipolar disorder (BD) symptoms in primary care comparing two screening instruments: Mood Disorders Questionnaire (MDQ) and Hypomania Checklist (HCL-32). *Participants*. Adult patients presenting to their primary care practitioners for any cause and reporting current depression symptoms or a depressive episode in the last 6 months. *Methods*. Subjects completed MDQ and HCL-32, and clinical diagnosis was assessed by a psychiatrist following DSM-IV criteria. Depressive symptoms were evaluated in a subgroup with the Patient Health Questionnaire (PHQ-9). *Results*. A total of 94 patients were approached to participate and 93 completed the survey. Among these, 8.9% screened positive with MDQ and 43.0% with HCL-32. MDQ positive had more likely features associated with BD: panic disorder and smoking habit (*P* < .05). The best test accuracy was performed by cut-off 5 for MDQ (sensitivity = .91; specificity = .67) and 15 for HCL-32 (sensitivity = .64; specificity = .57). Higher total score of PHQ-9 was related to higher total scores at the screening tests (*P* < .001). *Conclusion*. There is a significant prevalence of bipolar symptoms in primary care depressed patients. MDQ seems to have better accuracy and feasibility than HCL-32, features that fit well in the busy setting of primary care.

## 1. Introduction

Bipolar disorder (BD) has an estimated lifetime prevalence rate between 2% and 6% when wider range of bipolar spectrum disorders is considered [1]. It is a complex mood disorder frequently associated with medical and psychiatric comorbidity and high suicide rate [2]. Suicidal risk in BD is esteemed to be 15–20 times higher than the general population, and self-harm ideation is reported by 79% of patients [3, 4]. Nevertheless, an average delay of 8–10 years from first onset of mood symptoms to a formal diagnosis of bipolar disorder occurs [5, 6]. Longitudinal researches show that a patient is euthymic for half of the time, while manic or hypomanic symptoms are present only in the 12%; in the rest of the time, a patient has depressive symptoms [7, 8]. Hypomanic symptoms are often perceived as egosyntonic, while it is depression that usually leads the patient to the physician [9]. Thus, any loss or lack of information on hypomanic symptoms increases the bias in favour of a diagnosis of depression [10].

Primary care is the health service entry point for the majority of people suffering from depressive disorders and therefore could play a key role in the detection and management of BD. Although prevalence of symptoms and diagnosis of BD is elevated in depressed patients of primary care, Frye et al. found that 78% of primary care physicians (PCPs) failed to detect or misdiagnosed BD [11, 12]. To improve recognition of BD, several rapid instruments have been developed in the last ten years, including the Mood Disorder Questionnaire (MDQ) and the Hypomania Checklist (HCL-32), now probably the most studied [13, 14]. Both the instruments are validated in psychiatric outpatients settings, and guidelines suggest their usefulness in secondary care, while few studies assessed the employ in primary care [15].

Gorski et al. first used MDQ in primary care to test the association with principal complaints of patients referring to their PCP and found that participants who did complain of anxiety and depression had higher incidence of positive MDQ scores (16.4%) [16, 17]. Das et al. found a lower prevalence (9.8%) in low-income patients and confirmed the association with anxiety and depression, worse quality of life, and more functional impairment [18]. Consistently, a French study reported a similar value of prevalence (8.3%) and a higher rate of positive screening among younger patients, separated, divorced, and unemployed [19]. Other studies conducted in specific populations, patients taking antidepressant, the presence of current indices of BD (depression, anxiety, and substance abuse) found higher prevalence rate ranging from 21.3% and 27.9% [20–22]. Only one study used HCL-32 in a primary care setting in comparison with another screening instrument, the Bipolar Spectrum Disorders Scale, reporting the 28.27% of test positivity with the HCL-32 [23, 24].

In addition, no studies directly compared the two instruments MDQ and HCL-32 in the primary care setting, while some comparisons are available in studies on psychiatric outpatient services. All of them showed similar overall screening qualities of the two tests, and sensitivity of HCL-32 was always slightly higher [25–30].

The objective of our study was to assess the prevalence of symptoms of the bipolar spectrum in primary care patients with current depression using the Mood Disorder Questionnaire (MDQ) and the Hypomania Checklist (HCL-32). In particular, we performed the first comparison between the two instruments in primary care.

## 2. Method

### 2.1. Setting and Participants

The present study was conducted in two primary care groups in Bologna in the first semester of 2011, located in the Borgo Panigale and Porto districts and included a total of 37 PCPs. In these groups, a psychiatric consultation-liaison project was implemented since 2001 and 2006, respectively. Furthermore, PCPs received a training about depressive disorder symptoms and DSM-IV diagnosis and criteria in 2009.

PCPs were asked to refer all patients aged 18 or more they visited in the study period and reported clinically relevant depressive symptoms or suffered from a depressive episode. Exclusion criteria were refusal to receive a psychiatric consultation or to participate in the study, inability to read or write, medical illness that would prevent completion of the interview, previous diagnosis of bipolar disorder, psychotic disorders, and mental retardation or cognitive impairment. 

### 2.2. Diagnosis of Mood Disorder

The diagnosis of current or past 6-month mood disorder was subsequently performed by a consultant psychiatrist of the Bologna Psychiatric Consultation-liaison Service. The psychiatrist followed DSM-IV criteria to formulate the diagnosis of mood disorder and was blind from results of MDQ and HCL-32. 

Patients referred by the Primary Care Group Porto also received the Patient Health Questionnaire (PHQ-9), a validated tool, composed by 9 item, corresponding to DSM-IV diagnostic criteria for major depressive episode [31]. The score is from 0 to 3 for each question; thus, the total score can range from 0 to 27. A higher score indicates greater depression: a patient score of 10 or greater suggests a diagnosis of MDD.

### 2.3. Bipolar Spectrum Symptoms Evaluation

Patients with a current or past (precedent 6 month) diagnosis of major depressive episode were asked to complete the MDQ and the HCL-32 to assess the prevalence of symptoms of the bipolar spectrum. Italian versions of the two instruments are available after studies of validation conducted in psychiatric outpatient services [14, 25, 32].

The MDQ is a self-report questionnaire composed by 17 questions: 13 yes/no items on the symptoms derived from the Diagnostic and Statistical Manual of Mental Disorder (DSM-IV) criteria and 4 questions about the cooccurrence of symptoms, levels of functioning, familiar history of BD, and previous diagnosis of BD. A positive MDQ screen is defined as endorsement of at least 7 or more symptoms items, cooccurrence of two or more symptoms, and moderate or severe impairment (MDQ standard cut-off) [13]. We also used another cut-off of 6 items without criteria of cooccurrence and functioning, as suggested by Hardoy et al. in the validation of the Italian MDQ for single-step studies in psychiatric outpatients [32].

The HCL-32 consists of 32 yes/no statements regarding a period when the patient remembers he was in a “high” mood. Items ask whether specific behaviours (e.g., “I spend more money/too much money”), thoughts (e.g., “I think faster”), or emotions (e.g., “my mood is significantly better”) were present in such a state. Higher scores reflect more severe hypomanic states. The HCL-32 standard cut-off is represented by the endorsement of at least 14 items or more. We also analyzed the cut-off of 12, proposed by Carta for single-step researches in an outpatient population [25].

In addition, data about sociodemographics, medical history, family history of psychiatric disorders, and current and past psychotropic medication were collected using specific forms.

### 2.4. Statistical Analyses

Patients screened as positive with the considered instruments were compared with those screened as negative. Chi-square test (*χ* ^2^) was used to compare the frequency of categorical variables between groups: frequency of positive and negative screenings and sociodemographic and clinical features. *T*-test was used to compare the means of continuous variables between two or more groups. Correlations between ordered variables as PHQ-9, HCL-32, and MDQ total score were assessed with Pearson linear correlation (*ρ*). The accuracy of the two screening instruments was calculated in terms of sensitivity and specificity for each possible cut-off point of the scales, considering as cut-off only the number of positive answers, in particular for MDQ no adjunctive criteria were comprised. Performance of the scales was assessed by means of the Receiver Operating Characteristic (ROC) Analysis [33]. Data were analyzed by using SPSS for Windows, version 17.0.

## 3. Results

### 3.1. Sociodemographic and Clinic Characteristics

Out of the 94 primary care attenders enrolled in the study, 93 completed the two questionnaires, and 67 received the diagnostic assessment of the consultant psychiatrist. The mean age of the participants was 49.1 (±15.1) years. The majority was female (72.3%) and had a secondary education level or higher (64.0%); forty-five point two percent were in a nonprofessional condition and in particular retired (18.0%), housewives (10.6%), unemployed (4.44%), and students (4.2%). Twenty point two percent of the sample was separated or divorced.

Eleven patients met criteria for BD II (11.7%). As many as 29 participants had a concomitant anxiety disorder (30.9%): 17 met criteria for generalized anxiety disorder (18.1%) and 12 for panic disorder (12,8%). Smoking habit was present in 30.0% of the sample, hypertension in 26.6%. Twelve patients (12.8%) reported the concomitance of two cardiovascular risk factors (we consider smoking habit, hypertension, dyslipidemia, obesity, and diabetes). About two-thirds of the patients were treated by PCPs with antidepressant drugs (67.0%); out of these, 74.2% were represented by Selective Serotonin Reuptake Inhibitors, 16.1% by Serotonin and Noradrenalin Reuptake Inhibitors, and 9.7% by other antidepressants including tricyclics. 

### 3.2. Diagnosis of BD and Screening Tests Scores

Patients meeting criteria for BD had a mean MDQ score of 6.18 (±1.85) and HCL-36 score of 15.36 (±4.90). Patients without diagnosis of BD had 3.10 (±2.77; *P* < .001) and 11.46 (±6.64; *P* = .069), respectively. Seven patients met criteria with a clinical diagnosis of BD and screened positive to both the two screening instruments, using standard cut-off. The MDQ positive and HCL-32 positive patients with confirmed or excluded BD diagnosis are summarized in Tables 1 and 2.

### 3.3. Symptoms Prevalence

Tables 3 and 4 summarize the endorsement rate of MDQ and HCL-32 items. In the sample affirmative responses to MDQ items ranged from 9.7% (“spending money got you or your family into trouble”) to 50.5% (“had much more energy than usual”); HCL-32 affirmative items responses ranged from 7.5% (“drink more alcohol” and “take more drugs”) to 76.3% (“feel more energetic and more active”). The symptoms elicited by 8 of the 13 items of the MDQ and 25 of the 32 items of the HCL-32 were more prevalent among participants screening positive than among those screening negative (*P* < .05). The proportion of participants who met the MDQ diagnostic criteria for bipolar spectrum was 8.6% for the standard cut-off and 23.7% considering the less restrictive cut-off of 6. The percentage of positive screening for history of hypomanic symptoms at the HCL-32 was 43.0% with 14 as cut-off and 55.9% for 12 or more items endorsed.

### 3.4. Characteristics Associated with MDQ+ and HCL-32+

The population identified by MDQ was part of the population that screened positive at the HCL-32. Characteristics associated to positivity to the two tests are presented in Tables 5 and 6. Using the cut-off standard of MDQ, we find clinical features related to positive screening: panic disorder (*P* = .029) and smoking habit (*P* = .028). MDQ-positive patients are more likely to be smokers and accordingly had a higher cardiovascular risk. The less restrictive cut-off (6) did not find correlations, except for current AD therapy. The HCL-32 positive patients were younger, more likely to have a high level of instruction, and they are more likely smokers (*P* < .05). There was no difference in gender between groups. With the standard cut-off (14), there was also a trend toward a higher incidence of positive screening in patients who were separated or divorced, but this did not reach statistical significance (*P* = .06).

### 3.5. Sensitivity and Specificity

**Figure 1:**
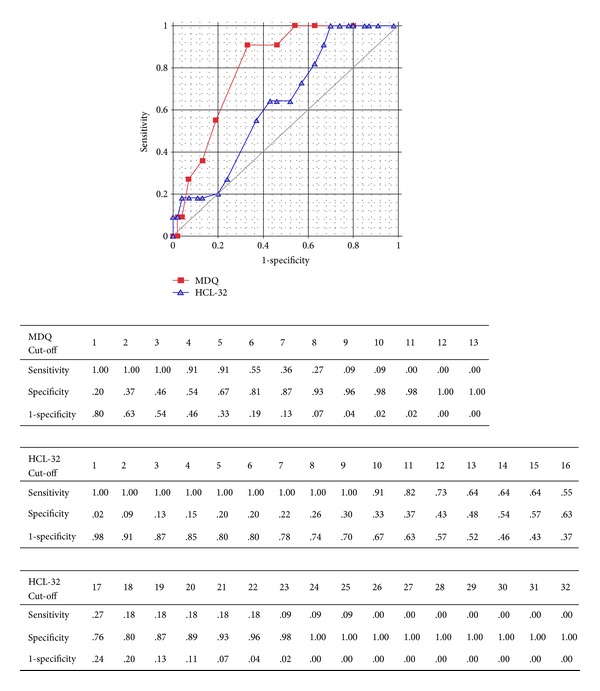
ROC Analysis of the performance of MDQ and HCL-32 in the sample.

Performances of MDQ and HCL-32 are illustrated by ROC analysis (Figure 1), with the report of sensitivity and specificity for each cut-off. The best accuracy of the test is given by cut-off 5 for MDQ (sensitivity = .91; specificity = .67) and 15 for HCL-32 (sensitivity = .64; specificity = .57). 

### 3.6. Relationship with PHQ-9

Analysis conducted in a subsample (*n* = 40) who received the PHQ-9 showed that higher PHQ-9 score correlated with both higher MDQ and HCL-32 scores (*ρ* = .316 − *P* = .036; *ρ* = .530 − *P* < .001, resp.). 

## 4. Discussion

In this study, we assessed the prevalence of symptoms ascribable to the spectrum of bipolar disorders through the use of two instruments meant for the screening of bipolar disorder (MDQ and HCL-32) in a clinical sample of primary care depressed patients. The two autosomministrated tests highlight the pattern of symptoms that can suggest an undiagnosed BD, but without diagnostic properties. Eleven patients (11,7%) met diagnostic criteria for BD, all of them are described as type II bipolar disorder, while the prevalence of bipolar spectrum disorder symptoms was very different between the two instruments. When standard criteria to establish the test positivity were considered, MDQ was positive in the 8.6% of the sample, whereas HCL-32 in the 43.0%.

Few studies were conducted in the primary care setting, and our MDQ positive rate appears to be consistent with values reported in previous researches recruiting not specific populations [18, 19]. On the contrary, primary care studies on selected patients (mood or other psychiatric disorder and/or in treatment with antidepressant drugs) reported higher values, between 21.0 and 27.9% [20–22], similar to those found in studies on psychiatric outpatients services [34, 35]. The lower prevalence of MDQ-positive reported in our sample may be due to the inclusion of patients with mild or subthreshold depressive symptoms, frequently observed in primary care. 

A higher rate of positive screening was found with the HCL-32. We can compare this result with only one primary care study using the HCL-32 in a population of patients with major depressive disorder. However, this research used a more restrictive cut-off (18) and reported 28.3% of test positivity [24]. Our finding seems to be more comparable to those obtained in secondary care and in very selected samples [27, 36, 37]. 

Considering the patients identified by the instruments, we found that MDQ positive patients were all included in the HCL-32 positive cases. HCL-32 test positivity identified some specific sociodemographic and clinical features: a younger age, a higher education level, and smoking habit. Consistently with previous studies [18, 34, 38], MDQ test positivity was associated only with clinical characteristics including panic disorder and smoking habit; in literature, these characteristics are both strongly related to BD [39, 40]. In addition, we found an association with MDQ-positive patients with at least two cardiovascular risk factors. These described correlations did not reach statistical significance using a less restrictive cut-off of the MDQ (6). Our data collected in primary care seem to indicate that MDQ test positivity detects a more specific population of depressed patients inside a wider group identified by HCL-32. 

The comparison in terms of psychometric proprieties seems to indicate a better accuracy of MDQ. We can also compare our findings with those obtained in one another Italian study that evaluated the two tests in a psychiatric setting [25]. Our results show lower values of sensitivity and specificity for both scales; however, MDQ sensitivity and specificity of the best performing cut-off remain similar to the results presented by Carta, while accuracy of HCL-32 is lower to a great extent. In particular, a less restrictive cut-off (5) of the MDQ showed better accuracy than the cut-off standard. The same trend of differences between MDQ and HCL-32 remains if we consider the other studies on the accuracy of the two instruments available in primary care [20, 24]. 

Considering these data, the rate of MDQ positive seems to be more consistent with the literature and with data on the prevalence of BD in depressed patients of primary care [11]. On the contrary, HCL-32 appears to be more sensitive in detecting subthreshold hypomanic conditions, identifying more than one-third of patients as cases, but this characteristic might not be compatible with the purpose in primary care of a close examination of all patients that screen positive. The population identified by MDQ and psychometric proprieties of the scale suggests higher accuracy of the instrument compared to HCL-32. Besides, MDQ shows a good feasibility: it is shorter compared to HCL-32 and a lower cut-off simplifies the scoring even further. These proprieties make MDQ accessible and usable in a primary care service and might aid PCP in order to better recognize BD in patients presenting depressive symptoms. 

An interesting finding emerged from the present study in a subsample. Patients who reported more hypomanic symptoms with a higher total score of the screening instruments correlated with higher total score for current depressive symptoms evaluated with the PHQ-9. We hypothesized that this result, reported also by Smith et al. [24], might be due to a subjective overestimation of previous well-being compared to the current depressive state, but it could also reflect the more severe condition of bipolar than unipolar depression [41]. As PHQ-9 is widespread in primary care, we could hypothesize a two-stage strategy to refine the mood disorders assessment. In particular, PCPs should also administer MDQ to patients reporting high PHQ-9 score. 

### 4.1. Limitations

The present study has several limitations. The initial assessment of mood disorder was done by PCPs, and there is the possibility of a misdiagnosis of depression. Subsequently the diagnosis of mood disorder was performed by a consultant psychiatrist following DSM-IV criteria, but without a structured diagnostic interview like the Structured Clinical Interview for DSM-IV Axis I Disorders (SCID-I) [42]. As a consequence, we admit a risk of selection of a highly heterogeneous sample and a possible under or overestimate of the diagnosis of BD; however, our symptoms prevalence and our values of sensitivity and specificity seem in line with previous literature on this topic [18, 19].

Furthermore, the sample was relatively small, and we gathered data to perform ROC analysis only for 67 patients; therefore, our results need to be interpreted with caution and further researches including studies with higher sample sizes are needed. 

Finally, we did not collect data about excluded patients and those that refused participation in the study or psychiatric consultation; however, the recruitment was performed in the busy setting of the primary care, and we chose to adopt a simple and easy procedure to avoid a supplementary work for PCPs. 

## 5. Conclusion

PCP is often the first health contact for assessment and treatment of patients with depressive symptoms. Despite programs of training and collaboration aimed to increase the PCP's ability to detect depressive disorders, differential diagnosis of a bipolar depression remains still a difficult and complex task. This background supports the use of specific instruments that can raise diagnostic accuracy of PCPs. 

In the present study, MDQ showed acceptable proprieties as screening instrument, with better psychometric characteristics using 5 as cut-off. MDQ appears to be more specific, easier and shorter than HCL-32, and it also takes little time to score. These features fit well in the busy setting of primary care, where PCPs have little time to dedicate to assessment. It cannot solve the diagnostic doubt between unipolar and bipolar depression, but it is the chance of having more patient's information during the visit to make correct therapeutic choices or to proceed in further investigations or refer to mental health services. 

## Figures and Tables

**Table 1:** MDQ-positive patients (standard cut-off*) and diagnosis of BD.

	BD−	BD+
MDQ−	52	9
MDQ+	4	2

*Standard cut-off: endorsement of at least 7 or more symptoms items, cooccurrence of two or more symptoms, and moderate or severe impairment.

MDQ: mood disorder questionnaire; BD: bipolar disorder.

**Table 2:** HCL-32 positive patients (standard cut-off*) and diagnosis of BD.

	BD−	BD+
HCL-32−	36	5
HCL-32+	20	6

*Standard cut-off: endorsement of at least 14 or more items.

HCL-32: hypomania checklist; BD: bipolar disorder.

**Table 3:** Frequencies of endorsed items to MDQ (standard cut-off*) and correlation with positive screening.

	Item	MDQ				
	endorsed, %	Positive, %	Negative, %	Chi-sq	df	*P*
(1) You felt so good/hyper that other people thought you were not your normal self or you were so hyper that you got in trouble?	16.1	37.5	14.1	2.955	1	ns
(2) You were so irritable that you shouted at people or started fights or arguments?	30.1	62.5	27.1	4.365	1	.037
(3) You felt much more self-confident than usual?	43.0	100.0	37.6	11.598	1	<.001
(4) You got much less sleep than usual and found you did not really miss it?	29.0	87.5	23.5	14.523	1	<.001
(5) You were much more talkative or spoke much faster than usual?	23.7	75.0	18.8	12.777	1	<.001
(6) Thoughts raced through your head or you could not slow your mind down?	29.0	75.0	24.7	8.977	1	.003
(7) You were so easily distracted by things around you that you had trouble concentrating or staying on track?	38.7	75.0	35.3	4.859	1	.028
(8) You had much more energy than usual?	50.5	75.0	48.2	2.095	1	ns
(9) You were much more active or did many more things than usual?	48.4	87.5	44.7	5.362	1	.021
(10) You were much more social or outgoing than usual; for example, you telephoned friends in the middle of the night?	12.9	12.5	12.9	0.001	1	ns
(11) You were much more interested in sex than usual?	22.6	75.0	17.6	13.758	1	<.001
(12) You did things that were unusual for you or that other people might have thought were excessive, foolish, or risky?	11.8	25.0	10.6	1.456	1	ns
(13) Spending money got you or your family into trouble?	9.7	12.5	9.4	0.080	1	ns

*Standard cut-off: endorsement of at least 7 or more symptoms items, cooccurrence of two or more symptoms and moderate or severe impairment.

MDQ: mood disorder questionnaire; Chi-sq: chi-square test; df: degrees of freedom; ns: nonsignificant.

**Table 4:** Frequencies of endorsed items to HCL-32 (standard cut-off*) and correlation with positive screening.

	Item	HCL				
	endorsed, %	Positive, %	Negative, %	Chi-sq	df	*P*
(1) I need less sleep	45.2	28.3	67.5	14.143	1	<.001
(2) I feel more energetic and more active	76.3	64.2	92.5	10.144	1	.001
(3) I am more self-confident	67.7	50.9	90.0	15.913	1	<.001
(4) I enjoy my work more	41.9	34.0	52.5	3.217	1	ns
(5) I am more sociable (make more phone calls, go out more)	58.1	41.5	80.0	13.870	1	<.001
(6) I want to travel and/or do travel more	37.6	24.5	55.0	9.018	1	.003
(7) I tend to drive faster or take more risks when driving	12.9	5.70	22.5	5.752	1	.016
(8) I spend more money/too much money	19.4	9.40	32.5	7.770	1	.005
(9) I take more risks in my daily life	19.4	15.1	25.0	1.433	1	ns
(10) I am physically more active (sport, etc.)	59.1	41.5	82.5	15.850	1	<.001
(11) I plan more activities or projects	59.1	35.8	90.0	27.662	1	<.001
(12) I have more ideas, I am more creative	55.9	30.2	90.0	33.083	1	<.001
(13) I am less shy or inhibited	35.5	15.1	62.5	22.378	1	<.001
(14) I wear more colourful and more extravagant clothes/make-up	18.3	3.8	37.5	17.358	1	<.001
(15) I want to meet or actually do meet more people	62.4	37.7	95.0	31.848	1	<.001
(16) I am more interested in sex, and/or have increased sexual desire	38.7	17.0	67.5	24.522	1	<.001
(17) I am more flirtatious and/or am more sexually active	28.0	9.4	52.5	20.991	1	<.001
(18) I talk more	38.7	18.9	72.5	20.448	1	<.001
(19) I think faster	41.9	18.9	27.5	26.928	1	<.001
(20) I make more jokes or puns when I am talking	36.6	22.6	55.0	10.291	1	.001
(21) I am more easily distracted	22.6	18.9	27.5	0.972	1	ns
(22) I engage in lots of new things	28.0	13.2	27.5	13.310	1	<.001
(23) My thoughts jump from topic to topic	24.7	13.2	40.0	8.791	1	.003
(24) I do things more quickly and/or more easily	57.0	35.8	85.0	22.467	1	<.001
(25) I am more impatient and/or get irritable more easily	30.1	18.9	45.0	7.398	1	.007
(26) I can be exhausting or irritating for others	20.4	13.2	30.0	3.954	1	.047
(27) I get into more quarrels	19.4	15.1	25.0	1.433	1	ns
(28) My mood is higher, more optimistic	68.8	52.8	90.0	14.676	1	<.001
(29) I drink more coffee	19.4	13.2	27.5	2.983	1	ns
(30) I smoke more cigarettes	15.1	5.7	27.5	8.503	1	.004
(31) I drink more alcohol	7.5	1.9	15.0	5.632	1	.018
(32) I take more drugs (sedatives, anxiolytics, stimulants)	7.5	5.7	10.0	0.617	1	ns

*Standard cut-off: endorsement of at least 14 or more items.

List of abbreviations: HCL-32: hypomania checklist; Chi-sq: chi-square test; df: degrees of freedom; ns: nonsignificant.

**Table 5:** Demographic and clinical characteristics according to threshold score on the MDQ.

	MDQ: standard cut-off*				MDQ: cut-off 6**			
	Positive	Negative	*F*/chi-square, df	*P*	Positive	Negative	*F*/chi-square, df	*P*
Gender, women, %	62.5	72.9	.402, 1	ns	63.6	74.6	1.022, 1	ns
Age, years: mean ± sd	48.4 ± 11.8	49.1 ± 15.4	.018, 1	ns	45.5 ± 13.9	50.2 ± 15.3	1.608, 1	ns
Education level, high, %	62.5	65.1	.833, 1	ns	77.3	60.9	2.019, 1	ns
Civil status, separated/divorced, %	25.0	20.0	.461, 2	ns	31.8	16.9	2.326, 2	ns
Occupation, nonprofessional condition, %	37.5	46.4	.247, 1	ns	40.9	47.1	.276, 1	ns
Current therapy, AD, %	62.5	67.1	.074, 1	ns	45.5	73.2	5.87, 2	.015
Panic attack disorder, %	37.5	10.6	4.757, 1	.029	18.2	11.3	.723, 1	ns
Smoking habit, yes, %	62.5	25.9	4.859, 1	.028	42.9	25	2.459, 1	ns
Cardiovascular risk factors, 2 or more, %	37.5	10.6	4.757, 1	.029	22.7	9.9	2.499, 1	ns

*Standard cut-off: endorsement of at least 7 or more symptoms items, cooccurrence of two or more symptoms, and moderate or severe impairment.

**Cut-off 6: endorsement of at least 6 or more symptoms items, with no adjunctive criteria.

List of abbreviations: MDQ: mood disorder questionnaire; Chi-sq: chi-square test; df: degrees of freedom; sd: standard deviation; ns: nonsignificant; AD: antidepressant.

**Table 6:** Demographic and clinical characteristics according to threshold score on the HCL-32.

	HCL-32: Standard cut-off*				HCL-32: cut-off 12**			
	Positive	Negative	*F*/chi-Square, df	*P*	Positive	Negative	*F*/chi-Square, df	*P*
Gender, women %	72.5	71.7	.011, 1	ns	71.2	73.2	.051, 1	ns
Age, years: mean ± sd	45.7 ± 13.6	51.6 ± 15.6	3.457, 1	ns	46.0 ± 14.2	52.9 ± 15.3	4.92, 1	.029
Education level, high %	76.9	55.8	5.273, 1	.037	78.4	47.5	9.34, 1	.002
Civil Status, separated/divorced %	30.0	13.2	5.638, 2	ns	25.0	14.6	2.124, 2	ns
Occupation, non professional condition %	42.5	48.1	.301, 1	ns	42.3	50.0	.560, 1	ns
Current therapy, AD %	57.5	73.6	2.674, 1	ns	44.2	19.5	6.343, 1	.012
Panic attack disorder %	12.5	13.2	.012, 1	ns	13.5	12.2	.035, 1	ns
Smoking habit, yes %	42.1	19.6	5.273, 1	.022	38.8	17.5	4.83, 1	.028
Cardiovascular risk factors, 2 or more %	20.0	7.5	3.176, 1	ns	15.4	9.8	.654, 1	ns

*Standard cut-off: endorsement of at least 14 or more items.

**Cut-off 12: endorsement of at least 12 or more items.

List of abbreviations: HCL-32: hypomania checklist; Chi-sq: chi-square test; df: degrees of freedom; sd: standard deviation; ns: non significant; AD: antidepressant.
